# Dexmedetomidine regulates the anti-oxidation and autophagy of adipose-derived stromal cells under H_2_O_2_-induced oxidative stress through Nrf2/p62 pathway and improves the retention rate of autologous fat transplantation

**DOI:** 10.3389/fphar.2024.1453938

**Published:** 2024-11-25

**Authors:** Zihao Li, Qing Wei, Yijun Li, Fangfang Yang, Chen Ke, Tian Li, Liqun Li, Zhongming Cai

**Affiliations:** Department of Plastic Surgery, The First Affiliated Hospital of Wenzhou Medical University, Wenzhou, China

**Keywords:** dexmedetomidine, adipose-derived stromal cells, anti-oxidative stress, autophagy, NRF2/P62 pathway, autologous fat transplantation

## Abstract

To investigate the protective mechanism of dexmedetomidine (DEX) on adipose-derived stromal cells (ADSCs) under oxidative stress model and its promotion effect on the retention rate of adipose granule transplantation by *in vitro* and *in vivo* experiments. The experiment was divided into control group, model group (ADSCs + H_2_O_2_+normal serum), DEX group (ADSCs + H_2_0_2_+DEX drug-containing serum), autophagy agonist group (ADSCs + H_2_O_2_+rapamycin (RAP)+normal serum), RAP + DEX group (ADSCs + H_2_O_2_+normal serum), RAP + DEX drug-containing serum), autophagy inhibitor group (ADSCs + H_2_O_2_+chloroquine (CQ)+normal serum), CQ + DEX group (ADSCs + H_2_O_2_+CQ + DEX drug-containing serum). HO-1, GSH-PX, SOD and CAT in ADSCs under oxidative stress model were measured. ROS fluorescence intensity and apoptosis ratio were detected. Expression of Nrf2, LC3-II/LC3-I and p62 were detected. *In vivo*, fat mixed with ADSCs or DEX -pretreated ADSCs was implanted subcutaneously in the lower back region of nude mice. Fat grafts were collected and analyzed at 2-, 4-, 6-, and 8-weeks post-transplantation. DEX pretreatment could reduce the expression of p62 to enhance the autophagy level of ADSCs under oxidative stress model. Additionally, cotransplantation of DEX-pretreated ADSCs with fat improved the long-term texture of fat grafts. DEX increased the fat graft survival and angiogenesis.

## Introduction

Autologous fat transplantation plays an increasingly important role in plastic and cosmetic surgery (Wolfe, 2022; Wu et al., 2021). As increase to repair parts of area, volume of an ideal soft tissue filling materials and important means, has been widely used in plastic surgery of facial soft tissue filler and facial treatment and other fields (Liu et al., 2022; Li et al., 2021). However, necrosis, apoptosis and absorption of a large number of adipocytes after transplantation due to ischemia and hypoxia are the main problems limiting the further development of adipocytes (Liang et al., 2023). Therefore, the key to autologous fat transplantation is to improve the survival rate after transplantation.

Autologous fat transplantation after ischemia reperfusion injury is a common pathophysiological process of plastic surgery clinical. It is currently believed that the long-term survival of adipocytes in this condition is the differentiation of new adipocytes from ADSCs, rather than the originally transplanted adipocytes (Schneider et al., 2023). However, during and after autologous fat transplantation, adipose stromal cells are exposed to substantial oxidative stress that negatively affects cell viability and graft survival (Taha et al., 2022; Lin et al., 2018). Therefore, how to prevent and reduce the oxidative stress injury caused by ischemia-reperfusion of autologous fat transplantation and improve the retention rate of autologous fat transplantation is the focus of plastic and aesthetic surgeons.

Autophagy is a “self-clearance” phenomenon widely existing in eukaryotic cells (Zhuang et al., 2021; Liu et al., 2021). Autophagy can regulate cell metabolism under normal conditions and remove damaged organelles and provide energy for repair after stress injury (Han et al., 2021). In the process of fat transplantation involving a number of mechanisms associated with autophagy, the results showed that higher autophagy theoretically can reduce cell oxidative stress damage, apoptosis (Lorzadeh et al., 2021), promote adipose stromal cells differentiate into fat cells and delay aging, inflammation by promoting paracrine function at the same time to improve the micro environment and promote angiogenesis (Yu et al., 2019; Zhou et al., 2021). Therefore, regulating autophagy is expected to be a means to improve the efficiency of fat transplantation.

In oxidative stress, Nrf2 further promotes the expression of HO-1, CAT, SOD and other antioxidant enzymes downstream of Nrf2 by combining with the antioxidant response element (ARE) sequence, so as to play antioxidant, anti-inflammatory and immune-regulating roles, thereby protecting tissues and cells from oxidative stress damage (Teng et al., 2023). However, excessive ROS is not only an activator of other stress response systems, including Nrf2, but also an activator of autophagy (Sun et al., 2023; Hou et al., 2023; Lan et al., 2023). Because the promoter region of p62, a key protein in autophagy process, also has an ARE binding site, Nrf2 can bind to ARE in the promoter region of p62 after entering the nucleus, and upregulate the expression of p62 to participate in the completion of autophagy (Riley et al., 2011).

Dexmedetomidine (DEX) is an α2 adrenergic receptor agonist with high selectivity, efficiency and other biological characteristics. DEX was originally used as sedation and analgesia for patients in intensive care units. Now it has been widely used in many fields, such as pre-anesthesia drug, general anesthesia adjuvant drug, postoperative analgesia and sedation (Jouybar et al., 2022; Baumgartner et al., 2022; Hu et al., 2020). With the recognition of ischemic preconditioning and postconditioning in clinical practice, drug preconditioning and postconditioning, due to its strong controllability, good repeatability, and small side effects, have more slight changes in hemodynamics and stronger tolerance of experimental subjects, which is more conducive to clinical application (Xiao et al., 2021; Miller et al., 2022). The mechanisms of DEX involved in organ protection include activation of cell survival kinase, regulation of apoptosis, and modulation of oxidative stress and inflammatory responses. Recent studies have highlighted its protective effects on various organs, including the brain, liver, and kidneys (Xiao et al., 2021; Miller et al., 2022; Ding et al., 2018), although reports on its protective effects during ischemia-reperfusion injury in autologous fat transplantation remain limited.

Therefore, we propose the following hypothesis: DEX regulates autophagy of ADSCs by activating Nrf2/p62 signaling pathway to protect ADSCs against oxidative stress injury under the oxidative stress model, thus promoting fat grafting survival.

## Methods

### Isolation and culture

ADSCs were isolated from adipose tissue obtained from randomly selected healthy individuals (BMI <25 kg/m^2^, females between ages 25 and 35, N = 6) undergoing lumbar abdominal ring suction surgery. All patients signed the informed consent for scientific research and publication. This study was approved by the Research Ethics Committee of Wenzhou Medical University (Issuing Number: 2023015). The adipose tissue from patients who underwent liposuction was dissected into smaller fragments in phosphate-buffered saline (PBS) and subjected to two washes with PBS. The intermediate layer of adipose tissue was then transferred to a 50 mL centrifuge tube. An equal volume of 0.2% type I collagenase was added, and the mixture was gently mixed through repeated pipetting. The centrifuge tube was subsequently placed in a temperature-controlled shaking incubator set at 37°C and 275 rpm for 45–60 min. Following digestion, an equal volume of high-glucose DMEM (supplemented with 10% FBS, 100 U/mL penicillin, and 100 mg/mL streptomycin) was added to terminate the enzymatic activity, and the solution was gently mixed. The resulting mixture was filtered through a 100-mesh filter to eliminate lipid components, followed by a filtration through a 200-mesh sieve to remove remaining impurities. The filtrate was aliquoted into 15 mL centrifuge tubes and centrifuged at 1,200 rpm for 10 min, with PBS washing repeated twice. If the precipitate contained significant amounts of red blood cells, the supernatant was discarded, and each tube received 3 mL of red blood cell lysis solution, followed by vortexing for 10 s and a 5-min incubation prior to a second centrifugation at 1,200 rpm for 5 min. The supernatant was discarded, and the resultant cell mass, representing the stromal vascular fraction (SVF), was resuspended in 2 mL of complete culture medium and inoculated into a cell culture flask at a density of 1 × 10^5 cells/cm^2^. The cultures were maintained in a humidified atmosphere with 5% CO2 at 37°C, with medium replacement occurring 48 h post-inoculation and subsequently every 48 h. Once the cells reached 90% confluence, they were subcultured and expanded, with subsequent experiments utilizing adipose stem cells subcultured to the third passage (P3). ADSCs were cultured in Human Adipose-derived Mesenchymal Stromal Cells Complete Medium with 450 mL Basal Medium For Cell Culture plus 50 mL Fetal Bovine Serum and Culture Supplement (catalogue no. HUXMD-90011, Oricel, Guangzhou, China).

### Characterization and the trilineage differentiation of ADSCs

ADSCs were identified using flow cytometry. Briefly, cells at passage 3 were harvested and 1 × 10^5^ cells were stained with specific fluorescent surface antibodies, including PE-conjugated anti-human CD29, CD31, CD44, CD45, CD90, and isotype IgG (1:100, Abcam, Cambridge, United Kingdom), using a flow cytometer (Beckman Coulter, CA, United States).

The trilineage differentiation potential of ADSCs was evaluated as previously described (Cai et al., 2023). Adipogenesis, osteogenesis, and chondrogenesis assays were performed using a specific differentiation medium (OriCell).

### Osteogenic differentiation

Osteogenic differentiation was induced by incubating adipose-derived mesenchymal stem cells in culture media supplemented with 10 mM β-glycerophosphate, 50 μM ascorbic acid, and 0.1 μM dexamethasone. The medium was replaced every 3 days for 3 weeks. After the completion of osteogenic induction, the old medium was removed and washed. Cells were stained by alizarin red and assessed with a phase-contrast microscope (Olympus).

### Adipogenic differentiation

Adipose-derived mesenchymal stem cells were induced in culture media supplemented with 0.5 mM 3-isobutyl-1-methylxanthine, 10 μM insulin, 200 μM indomethacin, and 1 μM dexamethasone. Three days after induction with medium A, medium B was added. After 24 h of induction, the old medium B was discarded, and an equal volume of fresh medium A was added to continue the induction. Alternate induction with medium A and medium B four times, then continue culturing with medium B for 7 days until the lipid droplets become larger and more rounded under the microscope. After induction, cells were incubated for 30 min in 0.5% (weight/volume) oil red O and assessed with a phase-contrast microscope (Olympus).

### Chondrogenic differentiation

For chondrogenic differentiation, the cells were incubated in culture media supplemented with 1 mM sodium pyruvate, 1% insulin–transferrin sodium selenite, 0.17 mM ascorbic acid, 0.35 mM L-proline, 1.25 mg/mL bovine serum albumin, 5.33 μg/mL linoleic acid, 0.1 μM dexamethasone, and 0.01 μg/mL TGFβ. The medium was replaced every 3 days for 4 weeks, and the cell morphology and growth were observed under a microscope. After induction, cells were assessed by toluidine blue and assessed with a phase-contrast microscope (Olympus).

### Cell counting kit-8 test

After observing that the cells were in the normal logarithmic growth phase, the cells were routinely digested and seeded into 96-well plates at a density of 5,000 cells per well. 96-well plates were incubated at 37°C in 5% CO2 incubator. After overnight incubation, the solution was changed, 100 ul of new complete medium was added to each well, and then the experimental H_2_0_2_ solution was prepared. Different volumes of 0.1 mol/L H_2_0_2_ solution were added to the corresponding Wells in the experimental group. The concentrations of H_2_0_2_ in each well were respectively 0.1mM, 0.25mM, 0.5 mM, 1.0 mM, 2.0 mM, and then the 96-well plates were incubated at 37°C and 5% CO2 for 0, 24, 48 h, respectively. 10ul Cell Counting Kit-8(CCK-8, GlpBio, CA, United States) was added to each well, and the culture was continued for 1 h. The absorbance value of each well was measured at OD450 nm of microplate reader.

ADSCs in logarithmic growth phase were collected and resuspended by trypsin digestion. Cells were digested conventionally and treated with 5,000 cells per well. The density of cells was seeded into a 96-well plate. Five rewells were set up in each group, and the observation time was set as 0h, 12 h, and 24 h. The model group (H_2_O_2_) and different concentrations of DEX groups (H_2_O_2_+DEX) were set. The model group was (1.0 mM H_2_O_2_ treatment for 24 h), and the drug groups were treated with DEX at concentrations (0.2 μM, 0.4 μM, 0.6 μM, 0.8μM and 1.0 μM for 0 h, 12 h, 24 h) after 1.0 mM H_2_O_2_ treatment for 24 h, and the cell viability was detected according to the above experimental procedures.

### Drug treatment

Once the oxidative stress model of ADSCs with optimal H_2_O_2_ concentration *in vitro* was established,and DEX stimulation concentration and duration are determined, the treatment groups were randomly divided into the following groups: control group, H_2_O_2_ group, H_2_O_2_ +DEX group, H_2_O_2_ +CQ group, OGD + DEX + CQ group, OGD + RAP group, and OGD + DEX + RAP group. The final concentration of 2 μg/mL chloroquine (CQ) or 10 nmol/L rapamycin (RAP) effectively inhibited or enhanced the activation of autophagy as evidenced by LC3 expression, respectively. The experiment was biologically replicated three times.

### Flow cytometry analysis

The working solution of fluorescein sodium dichloroacetoacetate (DCFH-DA) was prepared, and the final concentration was 10umol/L. ADSCs in logarithmic growth stage were inoculated into 6-well plates, incubated at 37°C with 5%CO_2_ for 24 h, and cultured according to experimental method 2.4. The cell culture medium was discarded, and 1 mL of the prepared DCFH-DA working solution with a final concentration of 10umol/L was added to the 6-well plate, and the solution was incubated at 37°C for 30 min and washed twice with PBS to fully remove the probes that did not enter the cells. The cells were digested with trypsin, and the cell suspension was collected in flow cytometry, centrifuged at 1,000 rpm for 5 min. The supernatant was discarded, and the cells were resuspended with 250ul PBS in each tube. The mean fluorescence intensity of the cells was detected by flow cytometry.

The number of apoptotic cells was measured by fow cytometry. Prepare a single cell suspension by adding 60% density of cells/well in a six-well plate. And then incubated for 24 h, the cells were digested with EDTA-free trypsin, After cell counting, 0.5 × 10^6^ cells were collected and centrifuged at 1500 rpm for 3 min. The supernatant was discarded and washed twice with pre-cooled PBS, the digested cells were then mixed and centrifuged in flow tubes. The cells were resuspended in 1 × bufer (Annexin V-APC/7-AAD (KeyGEN, Nanjing, China). Totally, 500 ul of this cell suspension was incubated with 5 μL Annexin V-APC and 5 μL 7-AAD dyeing solution at 4°C in the dark for 15 min. Finally, apoptosis was detected by flow cytometry (Beckman, CA, United States).

### Enzyme-linked immunosorbent assay (ELISA)

ADSCs in logarithmic growth stage were inoculated in 24-well plates and incubated at 37 C with 5%CO_2_ for 24 h. The cells were organized into groups according to drug treatment, with six replicates established for each group. Following incubation, supernatants were collected, and an ELISA kit (Abcam) was utilized in accordance with the manufacturer’s instructions to quantify the activities of glutathione peroxidase (GSH-PX) and superoxide dismutase (SOD), as well as the levels of catalase (CAT) and heme oxygenase-1 (HO-1).

### Western blot

Total protein was extracted with ice-cold RIPA lysis bufer (Beyotime) and PMSF (Phenylmethanesulfonyl fluoride) in a 100:1 ratio. For each sample, a quantity of 20 µg total protein per lane was separated by 10% SDS-PAGE at 80 V for 30 min, and 120 V for 40 min then transferred onto 0.2 μm Poly Vinylidene fluoride (PVDF) membranes (Millipalysisore, MA, United States) at 300 mA for 60 min in an ice bath. Then the PVDF membrane bound to the protein was taken out and blocked with 20 mL of QuickBlock™ Blocking Buffer (catalogue no. P0220, Beyotime, Shanghai, China) at room temperature for 15 min. The membranes were incubated overnight at 4°C with antibodies (Proteintech),containing Nuclear-factor-E2-related factor 2,Sequestosome 1, Microtubule-associated protein1Light Chain 3and Glyceraldehyde-3-phosphate dehydrogenase. The specific sources of antibodies used are shown in Table 1. The membranes were washed with PBST the next day, followed by incubation with horse radish peroxidase-conjugated goat anti-rabbit IgG secondary antibody for 1 h at room temperature. Protein bands were visualized by incubating the membranes with enhanced chemiluminescence reagent (Termo Fisher Scientifc). And the expression of indicated proteins were scaned by Alpha View software and quantifed by ImageJ software 1.51 (National Institutes of Health, Bethesda, MD, United States).

**TABLE 1:** Antibodies used for Western blots.

Antibody	Resource	Dilution ratio	Identifier
Nrf2	Proteintech	1:2000	16396-1-AP
p62	Proteintech	1:1,000	12143-1-AP
LC3	Proteintech	1:2000	81004-1-RR
GAPDH	Proteintech	1:1,000	10494-1-AP
Secondary antibody	Beyotime	1:5,000	A0277

### Real-time quantitative PCR

Total RNA was isolated by TRIzol Reagent (Termo Fisher Scientifc) from the frozen tissue samples or cell lines. cDNA was obtained from RNA using a PrimeScript RT Reagent Kit (TaKaRa, Dalian, China) according to the manufacturer’s protocol. Resultant products were amplifed using a SYBR Green PCR kit (TaKaRa, Dalian, China) and analyzed by ABI 7500 (Termo Fisher Scientifc).

### Immunofluorescence staining of cells

Cells were fixed in 4% paraformaldehyde in PBS for 20 min and permeabilized with 0.5% Triton X-100 in PBS for 15 min. These were then incubated with anti-Nrf2 (Abcam, 1:100), anti-p62 (Affinity, 1:100), anti-LC3 (Affinity, 1:100), and Alexa Fluor 488–labeled goat anti-rabbit IgG (H + L) (1:500, Beyotime) as secondary antibodies.

### In vivo autologous fat transplantation

BALB/c nude mice (male, 3–5 weeks old, weighing 16–18 g) were obtained from Zhejiang Vital River Experimental Animal Technology Co., LTD. (Zhejiang, China) and were kept in the SPF-class housing of Medical Animal Experimental Center of the First Affiliated Hospital of Wenzhou Medical University.

Adipose tissue was obtained from subcutaneous fat discarded during liposuction from an inpatient at the Department of Plastic Surgery, The First Affiliated Hospital of Wenzhou Medical University (Issuing Number: WYYY-IACUC-AEC-2023-001). Informed consent was signed with the patient before surgery.The adipose tissue was washed three times with pre-cooled PBS solution, centrifuged at 1,200 rpm at 4°C for 5 min, and the adipose tissue could be seen floating in the upper layer of the centrifuge tube. A small pipette was used to aspirate the grease on the top layer and the excess PBS, swelling anesthesia solution and residual blood in the lower tissue.

To determine the effects and investigate the mechanisms of DEX-pretreated ADSCs on Autologous fat transplantation, the adipose tissue granule was divided into 3 groups (n = 4 mice per group): control group, ADSCs group, and DEX-ADSCs group.Experimental units were allocated using RAND function. Sample size calculation: n = (t_0.05_ * 0.5)^2^/0.5^2^ = 4. BALB/c nude mice were anesthetized with 1% sodium pentobarbital (40 mg/kg weight, ip).In the control group, 500 μL of a mixture of fat and an equal volume of complete medium was injected. In the ADSCs group, a mixture of 500 μL fat and 10^7^ ADSCs was injected. In DEX-ADSCs group, 500 μL of a mixture of fat and 10^7^ dexmedetomidine pretreated ADSCs was injected. Briefly, fat was injected with a needle (1.2 × 38 mm, KDL, Shanghai, China), and The needle is filled with specimen fat beforehand, Three symmetrical points (upper right, lower left, and lower right) were selected on the back with the spine as the midline. A needle was used to create a subcutaneous tunnel from a distance, and 500 μL of fat mixture was injected into each point.When the mice wake up, make sure there is no problem, it means that the model is successful.At scheduled times (2, 4, 6, and 8 weeks) post-transplantation, mice were sacrificed with pentobarbital sodium (100 mg/kg weight, ip), and four randomly selected animals in each treatment group were euthanized and the grafts were harvested for subsequent analyses. The experimenters conducting the outcome assessment and the data analysis were blind to the group allocation.

### Graft weight measurements

We obtained a photograph of each graft and removed the residual and necrotic tissue with tissues of scissors and toothed tweezers. Adipose tissue mass was weighed using a microbalance.

### Histology analysis

The tissue was fixed with 4% paraformaldehyde, embedded in paraffin, sectioned by microtome and stained with hematoxylin and eosin (HE) and Masson staining. The overall integrity of the fat grafts was assessed by three blinded observers. The histological parameters were examined using a light microscope. The primary antibodies used for immunohistochemical staining were rabbit anti-human CD31 (Santa Cruz Biotechnology, Texas, United States) 、anti-human VEGF (Santa Cruz Biotechnology, Texas, United States) and anti-perilipin (Abcam). Three random fields were selected from each sample (n = 3/group). All measurements were performed using the ImageJ software.

### Statistical analysis

All values are presented as the mean ± SD. Normally distributed data were analyzed using t-tests or one-way analysis of variance (ANOVA). If data were not normally distributed, the Mann–Whitney *U* test was applied. In this study, *p* < 0.05 was considered statistically significant. GraphPad Prism (GraphPad Software, Inc., San Diego, CA, United States) was used for statistical analysis.

## Results

### Characterization of ADSCs

**FIGURE 1:**
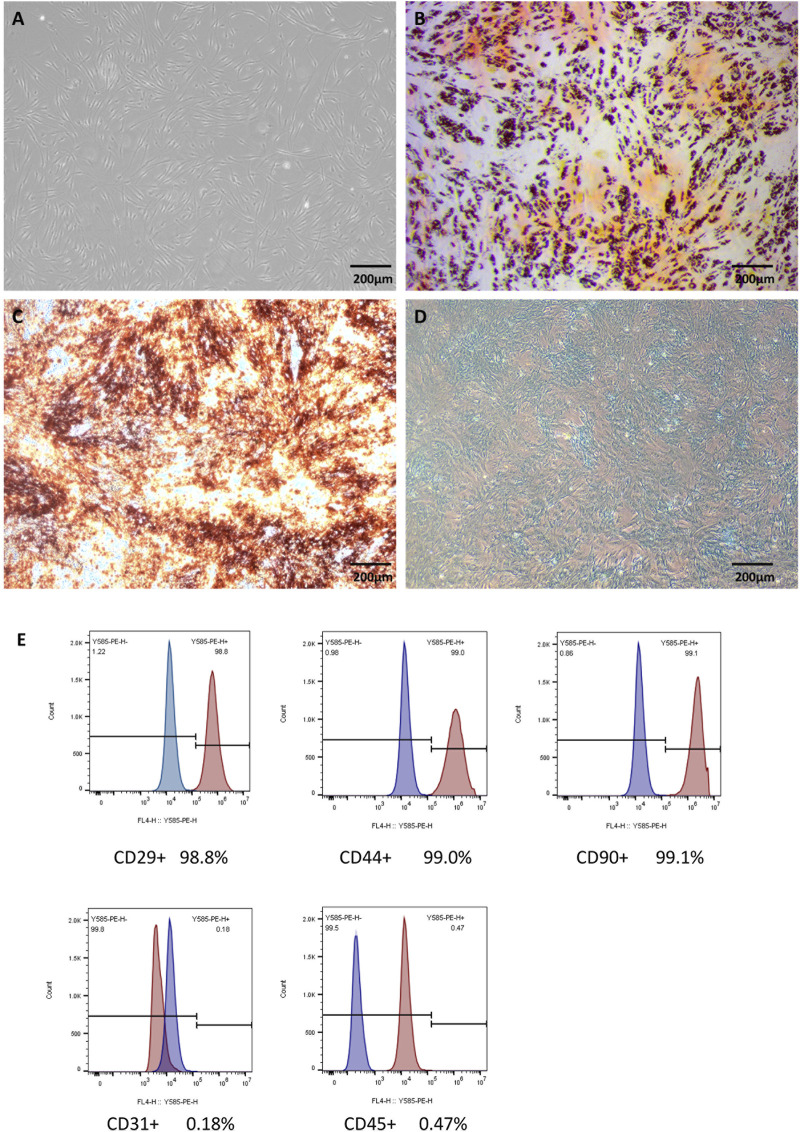
Characterization of ADSCs Legends: **(A)** The microscopic appearance of the 3rd generation of ADSCs. **(B)** ADSCs were stained with Oil red O after adipogenic differentiation. **(C)** Alizarin red staining after osteogenesis induction. **(D)** Alcian blue staining after induction. **(E)** Flow cytometry of cell markers in ADSCs.

Under the light microscope, scattered and polygonal adherent cells were observed at the bottom of the dish. When observed daily, the cells grew in the shape of long spindles, clonal colonies and swirls, and the morphology was relatively consistent (Figure 1A). The trilineage differentiation potential of ADSCs was assessed (Figures 1B–D). Flow cytometry demonstrated the immunophenotype of the cells that were positive for CD29, CD44, CD90 and negative for CD31, CD45 (Figure 1E).

### DEX improved the cell activity of ADSCs exposed to H2O2

**FIGURE 2:**
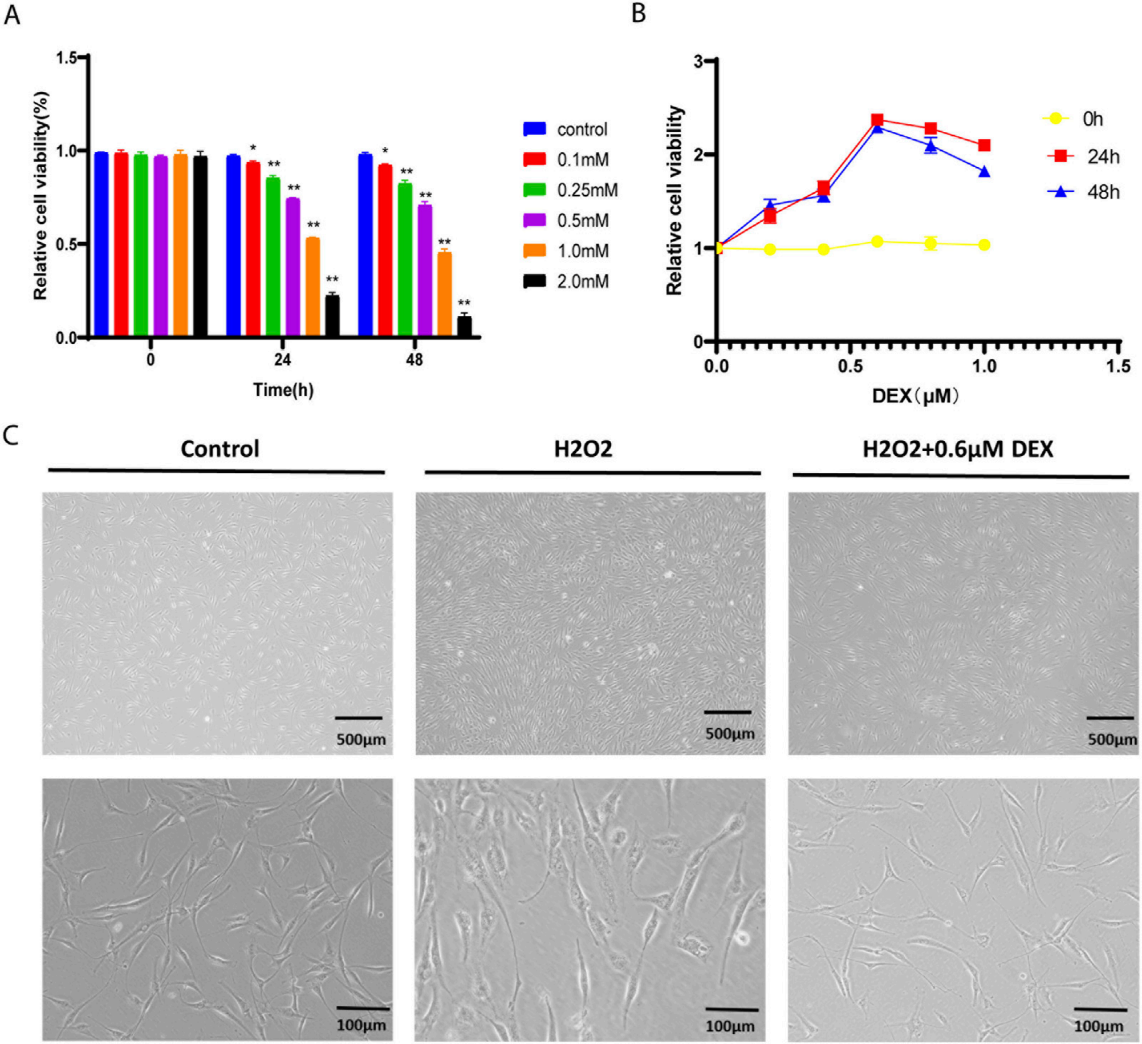
The oxidative stress model of ADSCs Legends: **(A)** Relative cell viability of hADSCs over time under different concentrations of H2O2. **(B)** Comparison of relative cell viability of different concentrations of DEX containing serum at different time points. **(C)** The morphology of ADSCs under oxidative stress model.

As was shown in Figure 2A, the activity of ADSCs decreased gradually with the prolongation of H_2_O_2_ stimulation time and the increase of concentration. After stimulation with 0.25 mM H_2_O_2_ for 24 h, the cell activity was significantly lower than that of the non-stimulated group (P < 0.01), the relative survival rate of cells approached IC50 after being treated with 1 mM H_2_O_2_ for 24 h, while the cell activity was more significantly decreased after being stimulated with higher concentration of H_2_O_2_(2.0 mM) for 24 h. According to the results of this experiment, we selected the stimulation concentration of 1 mM H2O_2_ for 24 h for the subsequent experiment, so as to clarify a series of biological changes that may occur in the cells during the initial stage of oxidative damage in normal ADSCs induced by H_2_O_2_.

As was shown in Figure 2B, in the oxidative stress model, ADSCs were treated with different concentrations of DEX at 0h, 24h, 48 h. Compared with the model group, there was no significant difference in the relative cell viability of ADSCs treated with 0.2 μM, 0.4 μM, 0.6 μM, 0.8 μM, 1.0 μM DEX at 0 h (*p* > 0.05). The relative cell viability of ADSCs increased after treatment with 0.2 μM, 0.4 μM, 0.6 μM DEX-containing serum for 24 h and 48 h, compared with the model group (*p* < 0.01). Compared with the 0.6 μM DEX group, the relative cell viability of the 0.8 μM and 1.0 μM DEX groups gradually decreased with the increase of drug concentration or the prolongation of action time. Therefore, the 24 h of action of 0.6 μM DEX with the highest activity was selected as the optimal concentration and time of drug-containing serum.

The effect of serum containing the optimum concentration of DEX on the morphology of ADSCs under oxidative stress model was observed by inverted microscope. As was shown in Figure 2C, compared with the control group, the dendrites of adipose stromal cells in the model group were significantly shortened and exfoliated after 24 h of 1.0 mM H_2_O_2_ stimulation. The dendrite retraction of adipose-derived stromal cells was prevented under the protection of 0.6 μM DEX. The results showed that 0.6 μM DEX could prevent the dendrite shortening of adipose stromal cells under oxidative stress.

### DEX could significantly increase the content of antioxidant enzymes in ADSCs under oxidative stress model

**FIGURE 3:**
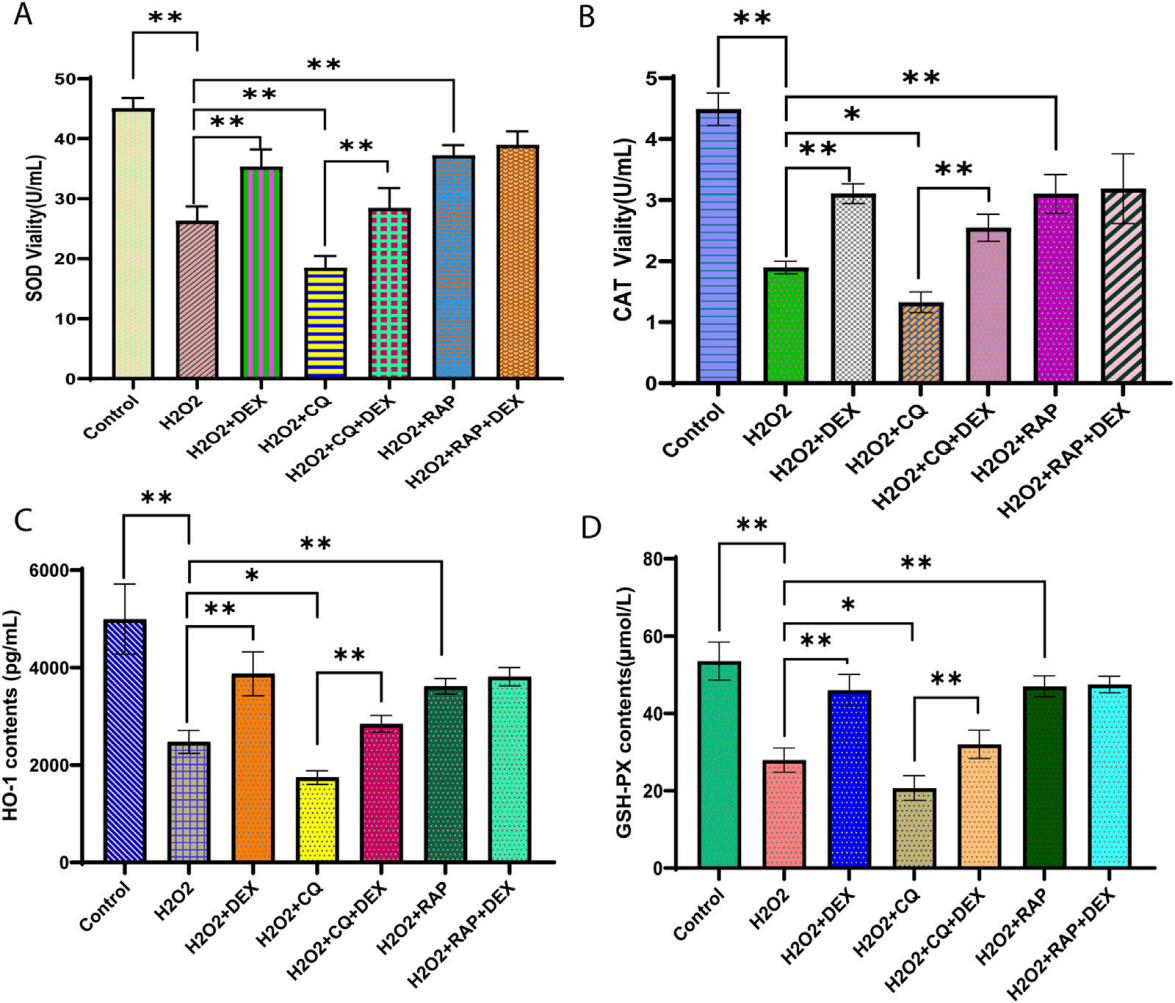
Dexmedetomidine inhibited the activity of SOD, CAT, HO-1 and GSH-PX in ADSCs under oxidative stress Legends: Relative concentration of **(A)** SOD, **(B)** CAT, **(C)** HO-1, and **(D)** GSH-PD in ADSCs treated with 0.6 μM DEX. *P< 0.05, **P< 0.01.

As was shown in Figure 3, compared with the control group, the activities of SOD and CAT in the model group were decreased, and the contents of HO-1 and GSH-PX were decreased (P < 0.01). Compared with model group, SOD and CAT activities, HO-1 and GSH-PX contents increased in DEX group (P < 0.01). In CQ group, SOD and CAT activities decreased, HO-1 and GSH-PX contents decreased (P < 0.01 P < 0.05). The activity of SOD and CAT increased, and the contents of HO-1 and GSH-PX increased in RAP group (P < 0.01). Compared with CQ group, SOD and CAT activities, HO-1 and GSH-PX contents increased in CQ + DEX group (P < 0.01). Compared with RAP group, SOD and CAT activities and HO-1 and GSH-PX contents in RAP + DEX group had no significant difference (P > 0.05).

### DEX could significantly reduce the ROS fluorescence intensity of ADSCs under oxidative stress model

**FIGURE 4:**
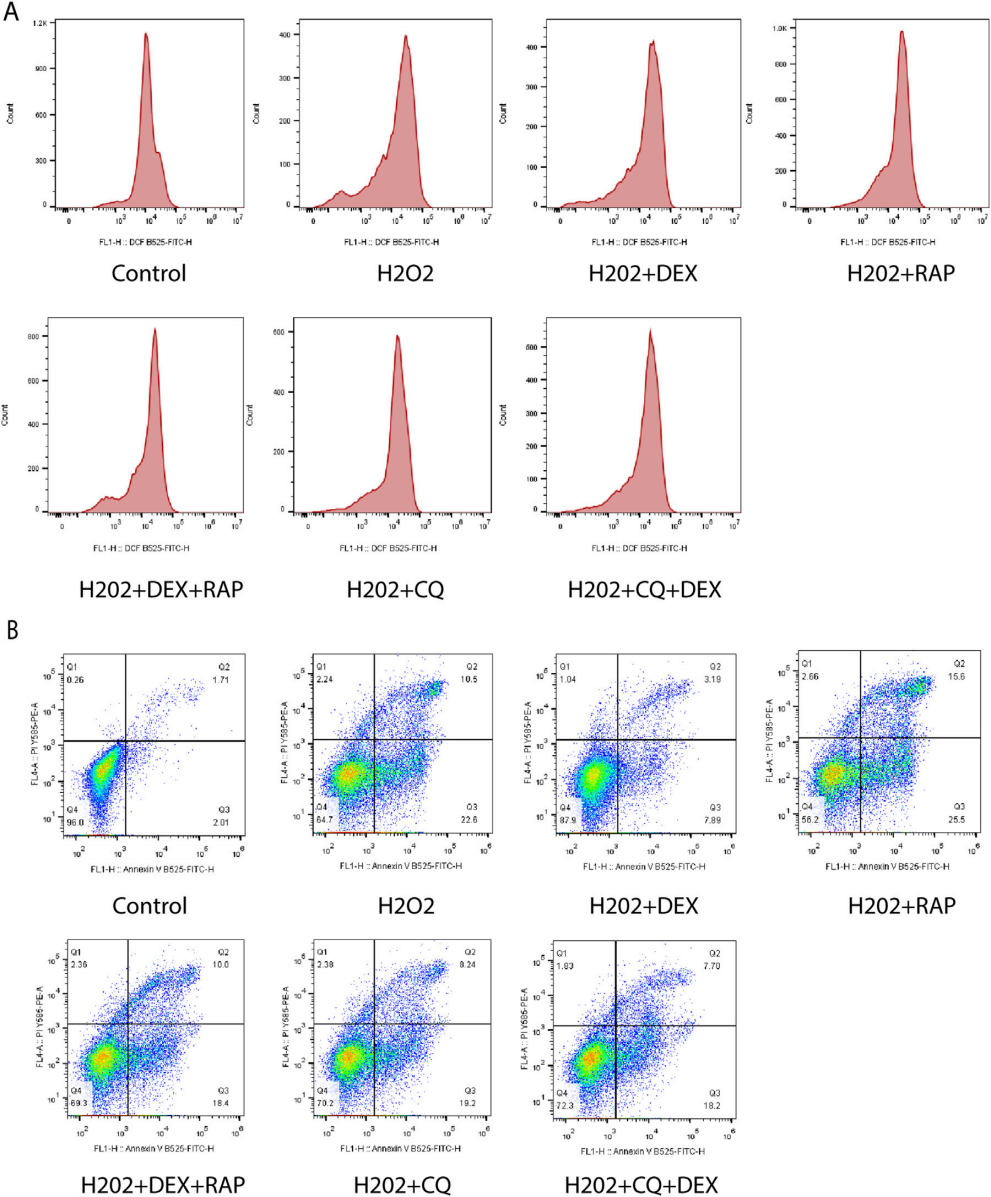
ROS and apoptotic cells of ADSCs under oxidative stress. Legends: **(A)** Effect of 0.6 μM DEX on ROS fluorescence intensity of ADscs under oxidative stress model. **(B)** Effect of 0.6 μM DEX on the apoptosis ratio of ADSCs under oxidative stress model.

As was shown in Figure 4A, compared with the control group, the ROS content of the model group was significantly increased (P < 0.01). Compared with the model group, the intracellular ROS content in DEX group decreased (P < 0.01), ROS content increased in CQ group (P< 0.01), and the levels of intracellular ROS in RAP group decreased (P < 0.01). Compared with the CQ group, the intracellular ROS content in the CQ + DEX group decreased (P < 0.01). Compared with RAP group, there was no significant difference in intracellular ROS content in RAP + DEX group (P > 0.05).

### DEX can significantly reduce the apoptotic ratio of ADSCs under oxidative stress model

As was shown in Figure 4B, compared with the control group, the proportion of apoptosis in ADSCs model group was significantly increased (P < 0.01). Compared with the model group, the proportion of apoptosis was significantly decreased in the DEX-containing serum group (P < 0.01), the percentage of apoptosis in CQ group increased significantly (P < 0.01), and the percentage of apoptosis in RAP group decreased significantly (P < 0.01). Compared with CQ group, the proportion of apoptosis in CQ + DEX group decreased (P < 0.05). Compared with RAP group, RAP + DEX group had no significant difference in the proportion of apoptosis (P > 0.05).

### Effect of DEX on Nrf2, LC3II/I and p62 protein expression in ADSCs under oxidative stress model

**FIGURE 5:**
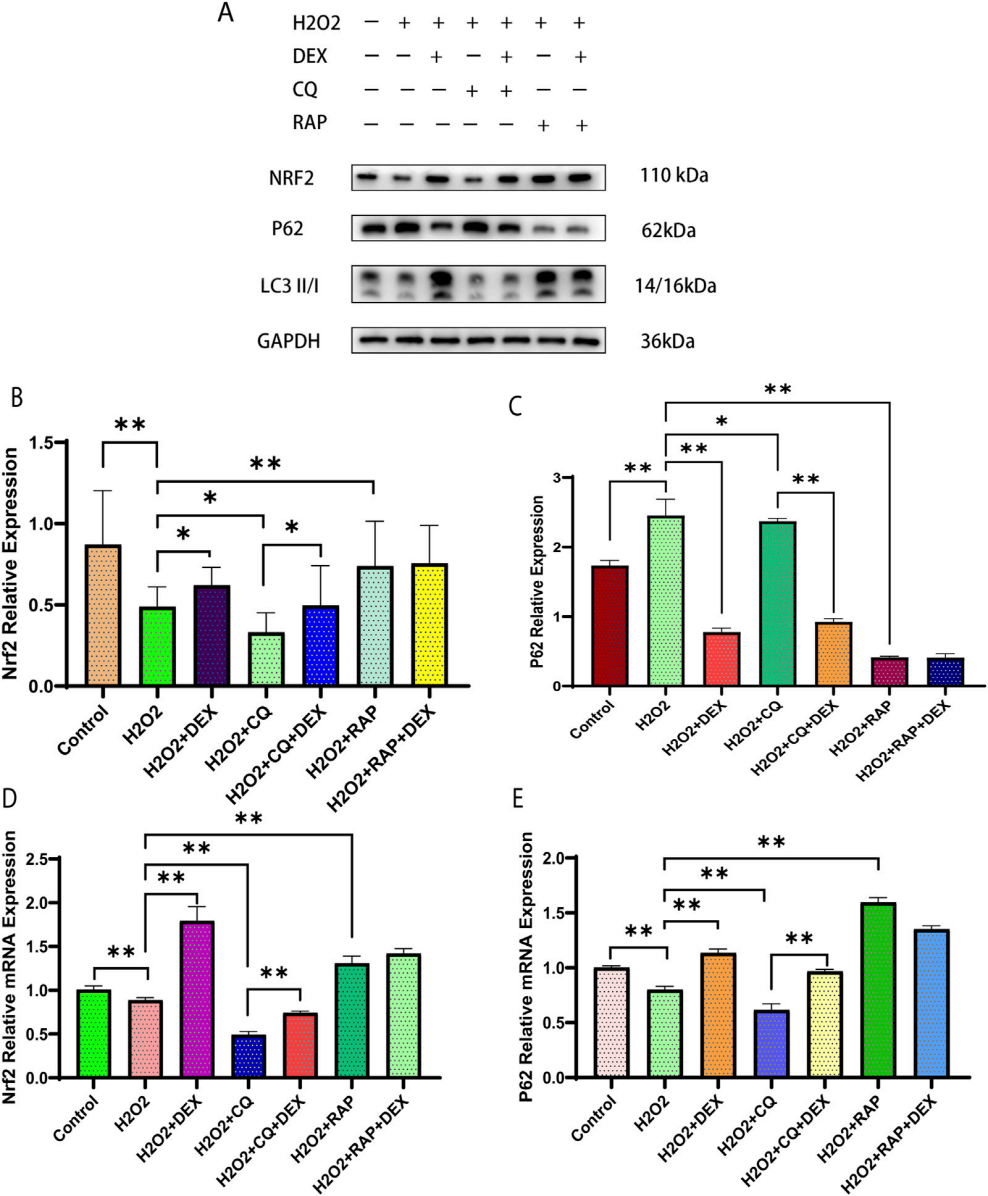
Effects of 0.6 μM DEX on expression of Nrf2, LC3II/I and p62 in ADSCs under oxidative stress model. Legends: Protein expression of Nrf2, LC3II/I and p62 were examined **(A)** and quantified by Western blotting **(B, C)** in ADSCs. **(D, E)** mRNA expression of Nrf2, LC3II/I and p62 in ADSCs. *P< 0.05, **P< 0.01.

As was shown in Figures 5A–C, compared with the control group, the expression levels of Nrf2 and LC3II/I proteins were downregulated and the expression level of p62 protein was upregulated in the model group (P < 0.01). Compared with model group, the protein expression levels of Nrf2 and LC3II/I were upregulated and the protein expression level of p62 was downregulated in DEX group (P < 0.01), the protein expression levels of Nrf2 and LC3 II/I were downregulated and the protein expression level of p62 was upregulated in CQ group (P < 0.01). The relative expression levels of Nrf2 and LC3II/I proteins in RAP group were upregulated, and the expression level of p62 protein was downregulated (P < 0.01). Compared with the CQ group, the expression of Nrf2 and LC3 II/I protein was upregulated in the CQ + DEX group, and the expression of p62 protein was downregulated (P < 0.01).

### Effect of DEX on Nrf2, p62 mRNA expression in adipose stromal cells under oxidative stress model

As was shown in Figure D–E, compared with the control group, the relative expression of Nrf2 and p62 mRNA in the model group was downregulated (P < 0.01). Compared with the model group, the relative expression of Nrf2 and p62 mRNA in BWJJ group was upregulated (P < 0.01), the relative expression of Nrf2, p62 mRNA in CQ group was downregulated (P < 0.01), and the relative expression of Nrf2 and p62 mRNA in RAP group was upregulated (P < 0.01). Compared with CQ group, the relative expression of Nrf2 and p62 mRNA in CQ + DEX group was upregulated (P < 0.01).

### DEX regulates fat grafting survival

**FIGURE 6:**
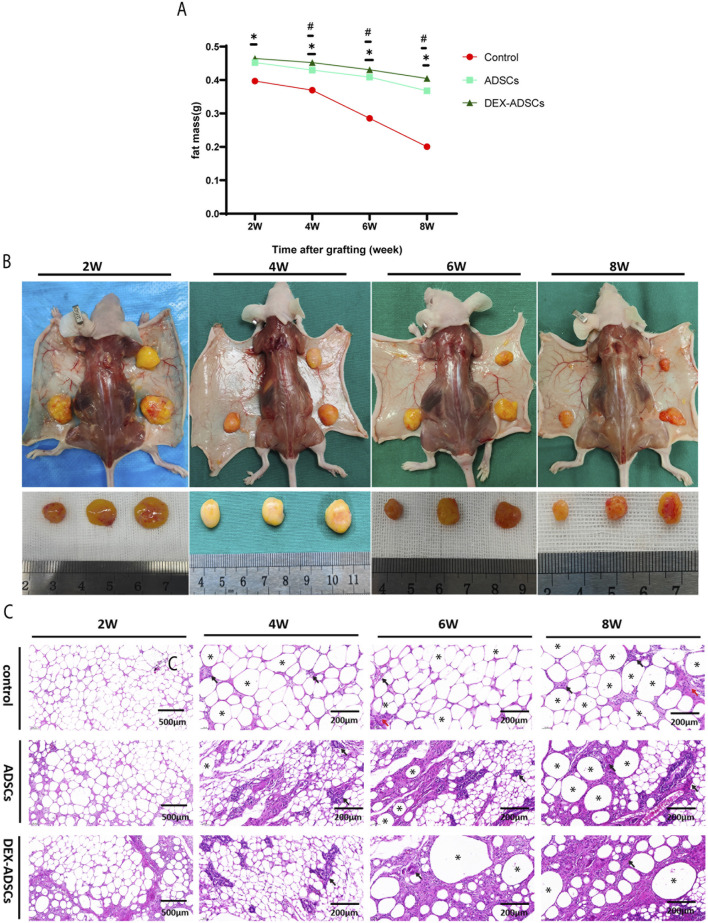
Effects of Dex on ADSCs co-transplantation on fat mass resorption Legends: **(A)** Fat mass at 2-, 4-, 6-, and 8-week post-transplantation. *p < 05. **(B)** Macroscopic images of the mice and fat grafts at 2-, 4-, 6-, and 8-week post-transplantation. **(C)** HE staining. Black arrow, representative image of monocytes infiltration, red arrow, representative image of fibrosis, *representative image of oil sacs.

After arriving at each time point, the transplanted cellulite was completely dissected and accurately weighed, and the mass change curve was drawn. It can be seen from the results that with the extension of time, the changes of cellulite mass in the ADSCs group and DEX-ADSCs group were significantly smaller than those in the control group, and from the fourth week, the decrease rate of cellulite mass in the ADSCs group was significantly higher than that in the DEX-ADSCs group (Figure 6A). Representative images of mice and grafts from each group are shown in Figure 6B.

**FIGURE 7:**
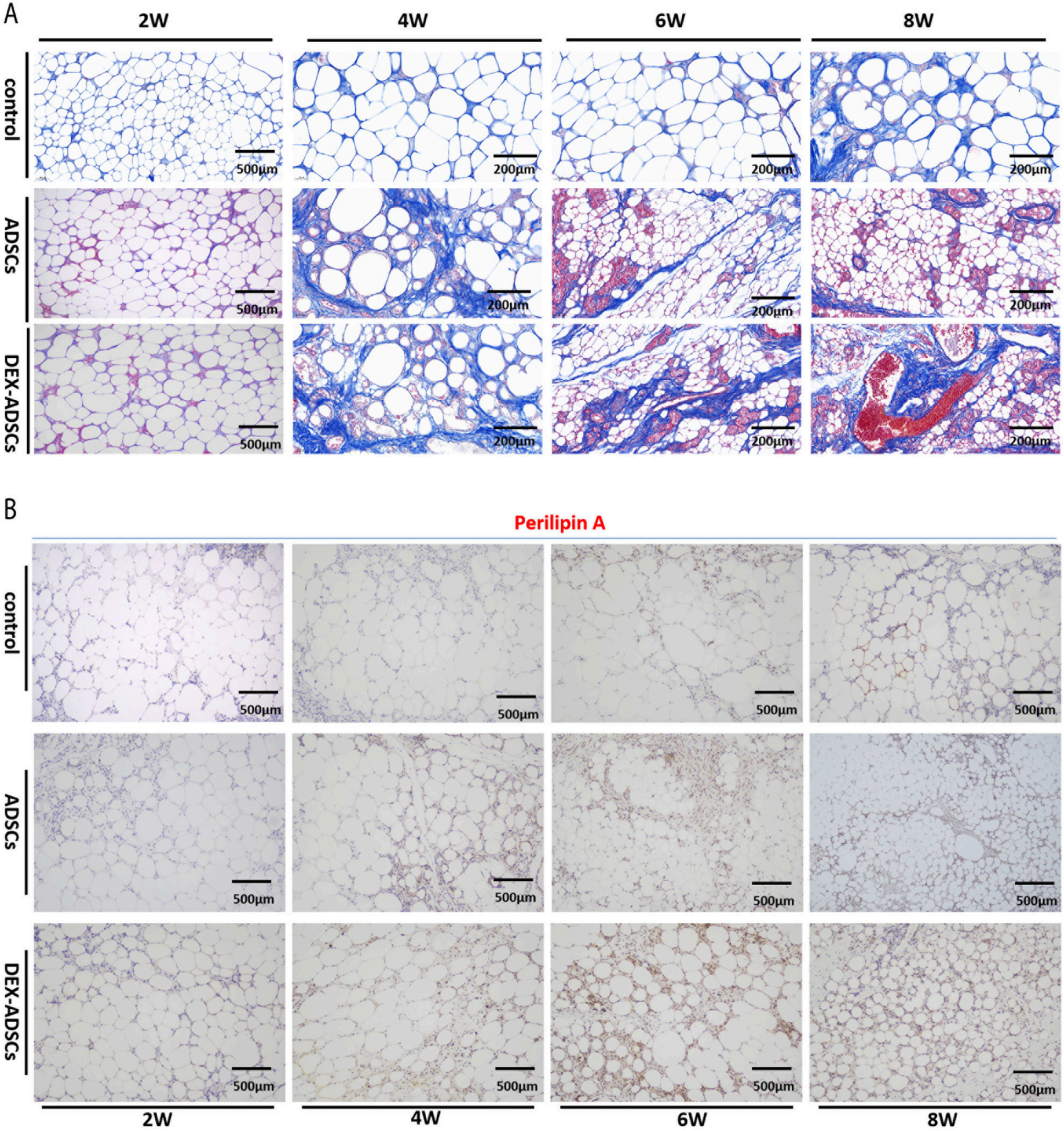
Effects of Dex on ADSCs co-transplantation Legends: **(A)** Masson staining and fibrosis analysis **(B)** Anti-perilipin A staining.

HE staining results (Figure 6C) showed that no obvious inflammatory cell infiltration was observed in the three groups at the second week. At the fourth week, a large number of monocytes infiltrated the adipocyte interstitium in the control group, while there was no significant change in the ADSCs and DEX-ADSCs groups. At 6 weeks, the infiltration of inflammatory cells in the control group was further aggravated, and interstitial fibrosis had begun. However, only a small amount of inflammatory cells were infiltrated in ADSCs and DEX-ADSCs groups. At 8 weeks, the control group showed more fibrosis and oil sac formation. The infiltration of inflammatory cells in the co-transplantation group was only slightly aggravated compared with that at 6 weeks, and there was no obvious interstitial fibrosis, even more mild in the DEX-ADSCs group (Figure 7A).

### Effect of DEX on functional status of adipose tissue after transplantation

Immunohistochemical staining results (Figure 7B) showed that perilipinA positive cells in lipid droplet membrane of cell co-transplantation group could be observed at the fourth week after fat transplantation, especially in DEX-ADSCs group. However, no perilipinA expression was found on the surface of lipid droplets in the control group. After 6 weeks, perilipinA positive lipid droplets gradually increased in cell co-transplantation group, especially in DEX-ADSCs group. At this time, a small amount of perilipinA was expressed on the surface of lipid droplets in control group. At week 8, perilipinA protein was expressed on the surface of almost all lipid droplets in the cell co-transplantation group, and the expression of Perilipin A protein was more uniform in the DEX-ADSCs group. The positive lipid droplets in the control group also increased, but the difference was still obvious compared with the cell co-transplantation group.

### Effect of co-transplantation of ADSCs and DEX-ADSCs on angiogenesis of transplanted cellulite

**FIGURE 8:**
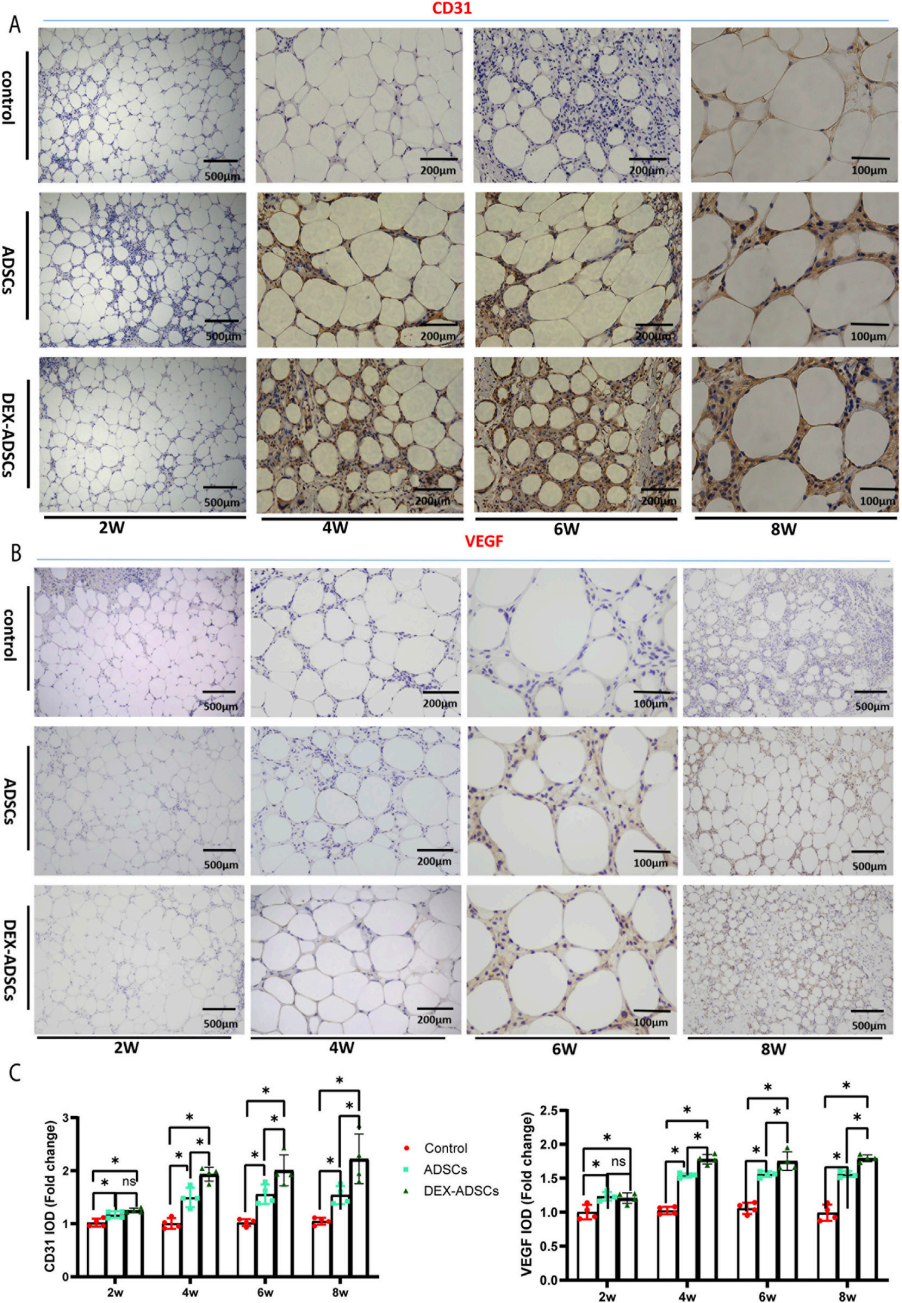
Effects of Dex on ADSCs co-transplantation Legends: **(A)** Anti-CD31 staining. **(B)** Anti-VEGF staining, **(C)** Integrated option density (IOD) analysis of CD31 and VEGF, *P< 0.05.

As was shown in Figure 8A, CD31 positive cells in the cell co-transplantation group increased significantly compared with the control group at 2 weeks (P < 0.05), but there was no significant difference between the two groups. At 4-, 6-, and 8- weeks, more CD31-positive blood vessles were found in the ADSCs group compared with the control group (P < 0.05). At the same time, more CD31-positive blood vessels were observed in the DEX-ADSCs group than in the ADSCs group (P < 0.05, Figures 8A, C).

It was found that VEGF was highly expressed in the DEX-ADSCs group than in the ADSCs group (P< 0.05) and in the control group (P < 0.05) at 4-, 6-, and 8- weeks, which verified the results similar to CD31 immunohistochemical staining (Figures 8B,C).

## Discussion

It is currently believed that the long-term surviving cells from autologous fat grafts are adipocyte precursors and ADSCs but not the mature adipocytes originally transplanted (Schneider et al., 2023). After transplantation, ADSCs were in a state of ischemia and hypoxia before restoration of blood supply (Lin et al., 2018). Our study investigated the protective mechanism of DEX on ADCSs under oxidative stress model *in vitro* and Nrf2/p62 pathway as the entry point.

We observed that H_2_O_2_ inhibited the proliferation activity of ADSCs in dosimetric and time-sensitive manner. After the cells were treated with 1 mM H_2_O_2_ for 24h, the relative survival rate of the cells approached IC50, while after the cells were stimulated with a higher concentration of H_2_0_2_ (2.0 mM) for 24 h and 48 h, A more pronounced reduction in cell activity was detected. Therefore, we selected 1 mM H_2_O_2_ stimulation concentration for 24 h to establish the oxidative stress model of ADSCs *in vitro*.

In the normal process of autophagy, p62 protein in the cytoplasm is continuously degraded with the completion of autophagy (Pantelis et al., 2023). When autophagy function is defective or autophagy activity is weakened, p62 protein will continue to accumulate in the cytoplasm. Therefore, p62 is one of the marker proteins reflecting autophagy activity (Filomeni et al., 2015). The relative expression of protein p62, LC3-I and LC3-II was detected by Western blot and the LC3 II/I ratio was compared. The results showed that compared with the blank control group, the LC3 II/I expression ratio of the model group decreased after the treatment of ADSCs with 1 mM H_2_O_2_ for 24 h. The relative expression of p62 protein increased, indicating that the autophagy level of ADSCs under oxidative stress model was low in the model group. Unlike previous studies applying DEX to cardiomyocytes *in vitro* (Borger et al., 2023)and kidneys (Zhou et al., 2023) *in vivo* to reduce oxidative stress injury, we treated ADSCs for 24 h. The results showed that the expression ratio of LC3 II/I increased, the relative expression of protein p62 decreased, and the level of autophagy increased. To further clarify whether DEX plays a protective role in ADSCs against oxidative damage by activating autophagy, we also observed the effects of autophagy inhibitor CQ group, CQ combined with DEX group, autophagy promoter RAP group. It was observed that the autophagy level of the inhibitor CQ group was lower than that of the model group, and that of the RAP group was higher than that of the model group. The autophagy level of the inhibitor CQ combined with BWJJ was higher than that of the CQ group.

Nrf2 is a key regulator of oxidative stress response, and there are distribution and transcription disorders in the process of oxidative stress in cells (Hu et al., 2022). Considering that autophagy adaptor protein p62 is a key protein in the process of cell autophagy, its protein expression is transcriptional regulation after Nrf2 activation into the nucleus (Chen, 2022). Therefore, first of all, we treated ADSCs with 1 mM H_2_0_2_ for 24 h. Compared with the blank control group, the mRNA expression of Nrf2 and p62 in the model group (H_2_0_2_ group) decreased, and the mRNA expression of Nrf2 and p62 in the DEX group was upregulated compared with the model group. By Western blot, we observed that compared with the blank control group, the expression of Nrf2 in the model histone was decreased, and the ratio of LC3II/I, a key protein in the formation of autophagosome, was downregulated, while the expression of p62, a specific substrate for autophagy, was increased. Compared with the model group, the expression of Nrf2 and LC3II/I were upregulated in the DEX group, these results were consistent with variation trend of expression of autophagy biochemical markers (p62/LC3-II) with activation of Nrf2 signaling pathway in blood cancer (Hussain et al., 2022). Downregulation of protein p62 expression. Because protein p62 binds to LC3 and participates in the formation of autophagosome membrane structure, insufficient protein p62 will lead to the formation of autophagosome block. Protein p62 in autophagy plays a “bridge” role in the process of connecting cytolysosome and autophagy substrates, suggests that protein p62 not only adjust the process of autophagy, simultaneously also unceasingly along with the completion of autophagy process degradation, this explains why in the process of the experiment p62 in protein level and gene level expression of appear inconsistent phenomenon, It may be because the enhanced autophagy activity leads to the downregulation of protein p62 along with the degradation of autophagy, and the dysfunction of autophagy leads to the continuous accumulation and upregulation of protein p62 in the cytoplasm (Profumo et al., 2022).

At the same time, we found that for the autophagy inhibitor CQ group, we used the autophagy inhibitor CQ to treat the ADSCs under the action of H_2_O_2_. Compared with the model group, we found that the expression levels of Nrf2mRNA and p62mRNA were downregulated, and the ratio of LC3 II/I, a key protein in the formation of autophagosomes, was downregulated. The relative expression of Nrf2 protein was downregulated and the relative expression of p62 protein was upregulated, indicating that the autophagy pathway was blocked and the nuclear entry of Nrf2 was downregulated. For the CQ + DEX group, compared with the autophagy inhibitor CQ group, the expression levels of Nrf2 mRNA and p62mRNA were upregulated at the same time, the ratio of LC3 II/I, a key protein in the formation of autophagosomes, and the relative expression of Nrf2 protein were upregulated. The relative expression of p62 protein was downregulated, indicating that the autophagy activity was enhanced, and the expression of p62 mRNA was upregulated by the nuclear entry of Nrf2 protein (Chen et al., 2022). These results indicated that under the oxidative stress model of ADSCs induced by 1 mM H_2_0_2_, DEX may regulate the autophagy level of ADSCs under oxidative stress by upregulating Nrf2-p62 pathway. In addition, we found that for the RAP + DEX group, when RAP + DEX combined treatment of ADSCs under the action of H_2_O_2_, compared with the autophagy agonist group RAP group, the results showed that the expression levels of Nrf2 mRNA and Nrf2 protein were upregulated, and the ratio of LC3 II/I, a key protein in the formation of autophagosomes, was downregulated. The downregulation of p62 mRNA level and upregulation of p62 protein relative expression level indicate that DEX can downregulate the excessive autophagy of ADSCs under the action of H_2_O_2_ caused by autophagy agonists, but the reason why Nrf2 protein and Nrf2 mRNA expression levels are not inhibited is unknown. Considering that Nrf2/p62 is a signaling pathway closely related to oxidative stress discovered in recent years, different from the classical signaling pathways such as Wnt and NF-kB, there is no mature inhibitor/activator of Nrf2-p62 pathway available for selection though ML385 have been showed to be applied in a prior study investingating the role of Nrf2 on survival and stemness of ADSCs (Hammad et al., 2024). With these questions, we hope to further explore in future studies.

Through further animal experiments, we found that DEX-ADSCs co-transplanted cellulite was superior to ADSCs group in terms of blood supply establishment and adipose tissue quality and activity, which was consistent with the prior study using DEX to alleviate acute stress-induced acute kidney injury by reducing oxidative stress in rats (Yang et al., 2024). Therefore, we hypothesized that DEX might stimulate adipose stromal cells to secrete more vasculogenic growth factors, thereby activating angiogenesis in transplanted adipose tissue. However, our observation is only preliminary and does not go deep into the molecular level to explore how DEX affects the secretion function of ADSCs by affecting a signaling pathway, which needs to be further studied.

## Conclusion

The present study investigated the protective role of DEX on ADSCs under oxidative stress conditions induced by H_2_O_2_, specifically through the Nrf2/p62 pathway. DEX was shown to enhance ADSC survival and autophagy levels, reduce apoptosis and ROS levels. The results indicated that DEX preconditioning helps preserve cell function and fat graft retention *in vivo*, facilitating improved fat quality and vascularization in autologous fat transplants. The findings suggested that DEX’s potential in enhancing fat graft longevity by activating the Nrf2/p62 pathway.

## Data Availability

The raw data supporting the conclusions of this article will be made available by the authors, without undue reservation.
